# Individual-Level Cyber-Risk Indicators and Patterns of Cyberbullying Involvement Among Korean Adolescents

**DOI:** 10.3390/healthcare14030376

**Published:** 2026-02-02

**Authors:** Yoewon Yoon, Kyoung Yeon Moon

**Affiliations:** 1 Department of Social Welfare, Dongguk University, Seoul 04620, Republic of Korea; yyoon@dongguk.edu; 2 Dharma College, Dongguk University, 30, Pildong-ro 1-gil, Jung-gu, Seoul 04620, Republic of Korea

**Keywords:** cyberbullying, perpetrator, victim, witness, overlapping roles, individual-level cyber-risk indicators, adolescents, media literacy, prevention

## Abstract

**Background/Objectives**: Although cyberbullying among adolescents has been widely studied, relatively little attention has been paid to the overlapping roles through which cyberbullying is experienced. This study reconceptualizes cyberbullying involvement by classifying perpetration, victimization, and witnessing into eight mutually exclusive involvement types, enabling systematic and non-overlapping comparison of adolescents’ experiences. The study further examines how engagement in individual-level cyber-risk indicators is associated with different patterns of cyberbullying involvement. **Methods**: The study analyzed nationally representative data from the 2022 Cyberbullying Survey conducted by the Korea National Information Society Agency, including 9693 students from elementary, middle, and high schools across South Korea. Individual-level cyber-risk indicators were assessed through multiple dimensions, including risky online behaviors, intensity of digital activity, peer environments, and awareness of harmful online behaviors. Multinomial logistic regression analyses were conducted to examine associations between individual-level cyber-risk indicators and the eight types of cyberbullying involvement. **Results**: Engagement in individual-level cyber-risk indicators was associated with increased odds of involvement in at least one cyberbullying type. Risky online behaviors and exposure to peers engaging in cyberbullying were linked to higher likelihood of both single and overlapping involvement patterns, whereas greater acceptance of harmful online behaviors was consistently associated with lower odds of victimization. **Conclusions**: These findings underscore cyberbullying as a relational and context-dependent phenomenon shaped by everyday digital practices and peer norms rather than isolated individual behavior. From a school social work perspective, the results support preventive, environment-focused interventions, including school-based media literacy education and institutionalized cyberbullying response systems, as promising strategies for reducing cyberbullying involvement among adolescents.

## 1. Introduction

Adolescents are increasingly engaged in social interaction and communication through digital platforms, as electronic social media technologies become deeply integrated into their daily lives and routines, thereby shaping alternative environments for peer relationships and social engagements [1,2]. The current generation of adolescents is accustomed to using cyberspace not only for social interaction and communication, but also for educational activities and knowledge acquisition [3]. While digital connectivity offers numerous benefits, it has also created new avenues for harmful behaviors. Among these, cyberbullying has emerged as one of the most pervasive and concerning issues, with rising prevalence across the globe and well-documented negative impacts on adolescents’ mental health, social adjustment, and academic engagement [4,5,6]. These developments highlight the urgent need to understand the dynamics of online peer interactions and the psychosocial risks they entail.

One of the most significant dangers confronting social media users—particularly young people—is online harassment, commonly referred to as cyberbullying [2,7]. Cyberbullying, typically defined as the intentional and repeated infliction of harm through digital channels, has drawn increasing scholarly and public attention amid the ubiquity of electronic devices and rapid technological advancement [8,9,10]. Unlike traditional bullying, cyberbullying is unconstrained by time or location, making it more pervasive and difficult for adolescents to escape [11,12,13]. Its cross-platform reach, anonymity, and potential for viral dissemination intensify the harm and complicate prevention efforts [14,15]. A growing body of research documents the detrimental consequences for adolescents, including poorer academic performance and increased dropout risk [16,17,18,19], as well as elevated mental health problems and psychosocial difficulties [8,18,20,21,22,23,24,25,26,27].

### 1.1. Experiencing Multiple Types of Cyberbullying

Adolescent involvement in cyberbullying is commonly categorized into three roles: perpetration, victimization, and witnessing [28,29,30,31]. Importantly, these roles are not mutually exclusive; research shows that adolescents may occupy them independently or simultaneously within a given period, reflecting the complex and overlapping nature of cyberbullying involvement [32,33,34]. The boundaries between perpetrators, victims, and witnesses are therefore fluid rather than fixed. For instance, some adolescents may remain solely perpetrators, while others may be involved in multiple roles within the same recall period, illustrating the co-occurrence of perpetration, victimization, and witnessing [15]. Overlapping experiences may escalate into a vicious cycle, reinforcing and amplifying bullying behaviors within shared online contexts. However, not all adolescents who experience victimization go on to engage in perpetration, indicating substantial heterogeneity in responses to cyberbullying. Emerging research suggests that individual-level psychological processes may help explain this divergence. In particular, online disinhibition—characterized by reduced perceived accountability, attenuated social cues, and weakened self-regulatory control in digital environments—may lower thresholds for retaliatory or reactive aggression following victimization [35]. Repeated exposure to online aggression may normalize hostility, reduce empathy, or facilitate moral disengagement, thereby increasing escalation risk among some adolescents, while difficulties in emotion regulation, heightened impulsivity, and perceived anonymity may further erode behavioral inhibition. At the same time, variation in coping strategies, social support, and normative beliefs may buffer against such escalation for others. Together, these mechanisms underscore the need to examine cyberbullying involvement not only in terms of role overlap, but also in relation to the psychological processes that shape how adolescents interpret and respond to harmful online experiences.

Although prior research has acknowledged that adolescents may experience cyberbullying in overlapping roles, comparatively few studies have examined the implications of treating these roles as mutually exclusive analytic categories. In particular, the role of witnessing remains underexplored, despite growing evidence that it may be intrinsically connected to perpetration and victimization or occur as a distinct and independent experience [34,36]. Witnesses occupy a central position in cyberbullying dynamics, shaping peer norms and influencing whether harmful behaviors are reinforced or challenged [37]. Despite Korea’s nationwide annual surveys that systematically monitor cyberbullying perpetration, victimization, and witnessing, intervention and educational efforts addressing the psychosocial impact of witnessing have remained limited and under-specified. This gap is particularly salient within the Korean context, where adolescents’ engagement with digital platform begins at an early age and peer relationships, learning, and leisure activities are deeply embedded in online environments [38]. High smartphone penetration and extensive use of mobile messaging, online gaming, and social media render cyberbullying not a discrete or episodic event but a relational experience that can be repeatedly enacted and diffused within digital space [39,40]. Under such conditions, roles of perpetration, victimization, and witnessing are unlikely to remain fixed or mutually exclusive within a given time frame; instead, they often co-occur or overlap within adolescents’ cyberbullying experiences. Moreover, cultural norms emphasizing harmony and relationship maintenance within peer groups may discourage direct intervention or confrontation, increasing the likelihood that witnessing manifests as tacit endorsement, conformity, or secondary involvement rather than neutral observation [41,42,43]. These sociocultural and digital characteristics underscore the importance of explicitly incorporating witnessing as an independent role and examining the distribution of role combination [36,44], making the present typological approach particularly relevant in the Korean setting.

Finally, Korea represents one of the few national contexts with a sustained, state-level infrastructure for monitoring youth cyberbullying using standardized survey instruments. This provides a robust empirical foundation for systematically comparing diverse involvement patterns. Accordingly, this study extends beyond a country-specific case analysis and serves as a theoretical test-bed for examining how cyberbullying involvement is structured in societies where digital interaction is fully integrated into adolescents’ everyday lives.

Building on evidence of its detrimental impacts, current research highlights the overlapping roles in cyberbullying—perpetrators, victims, and witnesses [34,45,46,47,48]. This reciprocal dynamic is amplified in online environments, where anonymity and disinhibition escalate hostile interactions across chats, games, and social networks [49,50]. Prior longitudinal and meta-analytic studies have documented cycles of role continuity and inversion; however, the present study focuses on the co-occurrence of cyberbullying roles within a single recall period, capturing how multiple forms of involvement coexist in adolescents’ recent experiences [51,52,53,54,55,56]. Empirical findings also confirm this overlap, with more than 20% of youth reporting both perpetration and victimization [57]. Witnesses also play a consequential role, in that their presence and responses shape the broader peer ecology. By standing up for victims in online settings, they can suppress bullying behavior and mitigate its harmful impact [58]. Crucially, such actions may also encourage other witnesses to strengthen their normative beliefs and willingness to support [59].

### 1.2. Individual-Level Cyber-Risk Indicators and Online Coexistence

Online platforms, as integral component of contemporary life, provide unparalleled opportunities for connection, information sharing, and social interaction. Yet their same features—accelerated communication, anonymity, and large-scale dissemination—also create conditions that intensify cyberbullying [8,15,60,61]. In cyberspace, an unspecified number of potential perpetrators, victims, and witnesses coexist simultaneously, fostering spatiotemporal proximity among them [20]. The anonymity of online environments emboldens individuals to engage in behaviors they might avoid offline, while weak formal and informal regulation reduced protection and heightens exposure to multiple forms of bullying [61,62].

For children and adolescents, online interaction constitutes a form of cyber coexistence in which risk is not confined to isolated behaviors but accumulates through repeated exposure, learning, and reinforcement within digital environment [63,64]. In this study, individual-level cyber-risk indicators are conceptualized as a multidimensional context encompassing risky online behaviors, normative attitudes toward cyberbullying, intensity of digital activity, and peer environments that collectively shape cyberbullying involvement. Risky behavior such as chatting with strangers expand exposure in anonymous and open platforms, increasing vulnerability to victimization while also heightening the likelihood of impulsive or retaliatory perpetration [61]. Repeated exposure to violence or explicit content can normalize aggression, reduce sensitivity to harm, and even encourage imitation, thereby weakening moral boundaries around cyberbullying [65,66]. Limited digital literacy further compounds these risks, as some adolescents lack the skills to manage online conflict safely or come to perceive cyberbullying as a routine aspect of digital life [67]. Extended screen time amplifies these processes by increasing cumulative exposure and eroding social and emotional self-regulation in loosely regulated online spaces [68]. Finally, peer networks exert a powerful influence: consistent with social learning theory [69], adolescents are more likely to imitate observed behaviors, and affiliation with peers who engage in cyberbullying can foster conformity, escalate aggression, and increase the likelihood of transitioning into multiple involvement roles, including perpetration, victimization, and witnessing [70]. Taken together, these dynamics highlight the online coexistence while an indispensable feature of contemporary adolescent life, generates distinct and cumulative risks. Understanding how the structural and cultural features of cyberspace interact with individual vulnerabilities is therefore critical for designing effective prevention and intervention strategies. Prior research has largely approached cyberbullying through a perpetrator-victim dichotomy or treated overlapping experiences as a single undifferentiated group, limiting comparative insight into how different role configurations emerge. Addressing this gap, the present study disaggregates perpetration, victimization, and witnessing into mutually exclusive involvement types and examines how each pattern is differentially associated with individual-level cyber-risk indicators. By doing so, cyberbullying is reconceptualized not as a fixed individual trait but as a relational experience shaped by conditions of online coexistence, offering a more nuanced framework for understanding adolescent cyberbullying and informing context-sensitive prevention efforts.

### 1.3. Current Study

This study advances the literature on cyberbullying in several ways. First, it analyzes nationally representative data on Korean children’s and adolescents’ online usage patterns and their encounters with cyberbullying, addressing the lack of comprehensive studies despite the annual release of such data. Second, the study broadens its scope beyond perpetrators and victims to include witnesses, a group whose exclusive impact has been largely overlooked, whether in combination with other roles or as stand-alone observers [71,72]. Third, we examine eight mutually exclusive categories of involvement among Korean youth—perpetration only, victimization only, witnessing only, perpetration with victimization, perpetration with witnessing, victimization with witnessing, involvement in all three roles, and non-involvement—to capture the complexity of overlapping and exclusive experiences. These eight-type classification captures all possible combinations of perpetration, victimization, and witnessing while enabling non-overlapping comparisons across involvement experiences. Finally, the study examines individual-level cyber-risk indicators and features of online coexistence in Korea as contextual correlates, assessing their associations with adolescents’ membership across distinct cyberbullying involvement patterns.

The current study advances hypothesis grounded in social learning theory and the online disinhibition effect; higher levels of individual-level cyber-risk indicators are hypothesized to be associated with a great likelihood of adolescents’ involvement in overlapping and mutually exclusive cyberbullying roles (e.g., simultaneous perpetration and victimization), compared with adolescents who are not involved in cyberbullying.

## 2. Materials and Methods

### 2.1. Participants and Procedures

The current study used data from the 2022 Cyberbullying Survey conducted by the Korea National Information Society Agency (NIA) [73]. This annual survey, now in its 10th edition, has been conducted since 2013 to examine the experiences and perceptions of cyberbullying among children and adolescents, with the goal of informing evidence-based prevention and response policies. Data were collected between 16 September and 24 November 2022, using a combination of in-person and online survey methods. The survey employed a stratified multistage probability sampling design to ensure national representativeness across regions, school levels, and demographic characteristics. Sampling was first stratified according to the national population structure by geographic region and school level, encompassing 19 strata across 17 cities and provinces and including both general and specialized high schools. Within each stratum, schools were selected as primary sampling units, followed by the selection of classes as secondary sampling units, with all students in selected classes surveyed using a clustered sampling approach. The final analytic sample comprised 9693 children and adolescents enrolled in elementary (grades 4–6), middle, and high schools across south Korea, included 3401 students from 142 elementary schools, 3229 students from 125 middle schools, and 3063 students from 145 high schools. This study represents a secondary analysis of previously collected data. The authors had no direct contact with participants, and no new data were collected for the purpose of this study. All data were fully anonymized prior to access, and individual participants cannot be identified. Accordingly, this study was exempt from Ethics Committee and Institutional Review Board approval.

### 2.2. Measures

#### 2.2.1. Cyberbullying Experiences

Cyberbullying perpetration was assessed using eight items measuring engagement in specific behaviors during the previous 12 months, including “insulting or upsetting someone in cyberspace,” “continuing to send e-mails, messages, or visit blogs or social media pages of someone who dislikes me and leaving comments or photos,” and “sending sexually suggestive posts, photos, or videos through computers/smartphones to someone who I knew would dislike it.”, and “being prevented from leaving internet chat rooms or smartphone messaging apps and being insulted or prevented from participating in conversations.” Responses were originally recorded on a four-point frequency scale ranging from 0 (none) to 3 (almost every day). Consistent with prior national analyses and policy reports using the same dataset, all items were recoded into binary indicators (0 = no, 1 = yes) and summed to create a composite measure, which was subsequently dichotomized as 0 (no perpetration) or 1 (any perpetration experience within the past year). This dichotomization strategy aligns with the study’s analytic focus on role participation and role combinations, rather than the intensity or frequency of individual behaviors. Given that the primary outcome of interest was the configuration of perpetration, victimization and witnessing experiences into distinct involvement types, using experienced-based indicators allowed for clearer classification and comparison across mutually exclusive categories.

Cyberbullying victimization was measured using eight items parallel to the perpetration scale, assessing experiences of being targeted by specific cyberbullying behaviors during the previous 12 months. Sample items included “being insulted or upset in cyberspace,” “having false or fabricated stories spread about me in cyberspace,” “receiving sexually suggestive posts, photos, or videos through a computer/smartphone, even though they knew I would dislike it,” and “being prevented from leaving internet chat rooms or smartphone messaging apps and being insulted or prevented from participating in conversations.” Responses were coded on the same four-point frequency scale as perpetration. Consistent with the analytic strategy, all items were recoded into binary indicators (0 = no victimization vs. 1 = any experience of victimization), summed, and dichotomized to indicate the absence (0) or presence (1) of any victimization experience within the past year.

Witnessing cyberbullying was assessed using a single item asking whether participants had observed cyberbullying incidents involving others during the previous 12 months. Response options distinguished between observing perpetration, victimization, both perpetration and victimization, or no exposure. For analytic purposes, this variable was recoded into a binary indicator (0 = no witnessing, 1 = any witnessing) to ensure conceptual and interpretive consistency with the perpetration and victimization measures and to facilitate classification into mutually exclusive involvement types.

#### 2.2.2. Individual-Level Cyber-Risk Indicators

Risky online behaviors. In the context of this cross-sectional study, risky online behaviors were conceptualized as correlates of cyberbullying involvement. Risky behavior in cyberspace was measured using five items capturing potentially unsafe online practices. These items included statements such as “I share my SNS or e-mail account passwords with my friends,” “I engage in sexual conversations with strangers in cyberspace,” and “I accept proposals from acquaintances I meet online (such as offline meetings and exchanging contact information or photos).” Participants rated their agreement with each statement on a four-point Likert scale ranging from 1 (strongly disagree) to 4 (strongly agree), with higher scores indicating greater engagement in risky online behaviors. A mean score across the five items was calculated to represent overall risky online behavior. Because the items capture heterogeneous forms of online risk behavior rather than a single narrowly defined construct, the internal consistency of the scale was modest (Cronbach’s *α* = 0.60). Accordingly, this measure was treated as an exploratory index reflecting the breadth of adolescents’ risky online practices, which likely contribute to the lower reliability.

Acceptance Attitude toward Cyberbullying. Acceptance attitudes were examined as an indicator of individual-level cyber-risk culture. Acceptance attitudes toward cyberbullying were measured using eight items assessing participants’ normative perceptions of harmful online behaviors, such as “Insulting or hurting someone’s feelings,” “spreading false or exaggerated stories about others,” and “sending sexually explicit posts, photos, videos, etc., without consent, even when aware that the recipient may dislike it.” Responses were coded on a four-point Likert scale (1 = strongly disagree to 4 = strongly agree). Items were reverse-coded so that higher values reflect greater acceptance of cyberbullying behaviors. The mean score across items was used to indicate overall acceptance attitudes. This scale demonstrated excellent internal consistency (*α* = 0.96).

Duration of Digital Activity. Digital activity duration was considered a contextual exposure variable potentially associated with cyberbullying involvement, without implying temporal precedence. Digital activity duration was measured using a single item assessing participants’ average daily internet use over the previous year, including both weekdays and weekends. Response options ranged from 1 (less than 1 h) to 5 (6 h or more), with higher scores indicating longer daily internet use. This variable was treated as continuous in the analyses to capture variability in digital exposure.

Harmful Friend. Exposure to peers who engage in cyberbullying was used to capture peer-contextual correlates of cyberbullying involvement, acknowledging that peer selection and influence processes cannot be disentangled in cross-sectional data. This measure was assessed with a single item asking participants whether they had friends who perpetrate cyberbullying. Response options ranged from none to more than seven friends. For analytic purposes, this variable was recoded into binary indicator, with 0 representing no such friend and 1 indicating the presence of at least one friend who engages in cyberbullying.

### 2.3. Covariates

Covariates included gender (0 = female, 1 = male), school level (1 = elementary school, 2 = middle school, 3 = high school), subjective academic achievement (1 = lower rank, 2 = lower-middle rank, 3 = middle rank, 4 = upper-middle rank, 5 = upper rank), and perceived household financial status (1 = lower rank, 2 = lower-middle rank, 3 = middle rank, 4 = upper-middle rank, 5 = upper rank). These variables were included to adjust for sociodemographic differences in cyberbullying involvement.

### 2.4. Analytic Approach

Analyses proceeded in four steps. First, adolescents’ cyberbullying experiences were classified into eight mutually exclusive involvement types: no involvement; single-role involvement (perpetration only, victimization only, or witnessing only); two-role overlap (perpetration–victimization, perpetration–witnessing, or victimization–witnessing); and involvement in all three roles (perpetration, victimization, and witnessing). Second, descriptive analyses were conducted to summarize sample characteristics across involvement types, including demographic variables (gender and school level) and key predictors of cyberbullying. Third, chi-square tests and one-way analyses of variance (ANOVA) were used to examine group differences in demographic and predictor variables across the eight involvement types. Finally, multinomial logistic regression analyses were conducted to estimate associations between individual-level cyber-risk indicators and cyberbullying involvement types, adjusting for covariates. The non-involved group served as the reference category, such that the models estimated relative risks of belongings to each involvement type compared to non-involvement. These modes were not intended to formally test all pairwise differences between single-role and overlapping-role groups, but rather to characterize patterns of associations relative to non-involved adolescents. All analyses were conducted using Mplus version 8.11.

## 3. Results

### 3.1. Descriptive Statistics

Table 1 presents the demographic characteristics of the sample and their distribution across the eight types of cyberbullying involvement. The sample consisted of 9693 adolescents, with an approximately equal gender distribution (50.3% female). Participants were evenly distributed across school levels, including elementary school (35.1%), middle school (33.3%), and high school (31.6%). The mean subjective academic achievement score was 3.36 (SD = 1.17, range = 1–5), indicating a moderate level of perceived academic performance. Perceived household financial status was also moderate 3.38 (SD = 0.89, range = 1–5), with relatively limited variability, suggesting a generally homogeneous socioeconomic profile within the sample.

With respect to individual-level cyber-risk indicators, participants reported a moderate level of risky online behaviors (M = 1.57, SD = 0.49, range = 1–4) and relatively low acceptance of cyberbullying behaviors (M = 1.34, SD = 0.57). On average, adolescents spent approximately 3.45 h per day online (SD = 1.22). Exposure to harmful peer environments was limited overall, with a mean score of 1.09 (SD = 0.37), indicating that most participants reported having few or no friends who engaged in cyberbullying.

Overall, 43.3% of participants reported involvement in at least one form of cyberbullying during the previous year, while the remaining 56.7% reported no involvement. The largest involvement group comprised adolescents who experienced victimization only (1 8.5%, *n* = 1793), followed by those reporting both perpetration and victimization (13.4%, *n* = 1299), underscoring the substantial prevalence of overlapping cyberbullying roles. Smaller portions reported perpetration only (3.8%, *n* = 371), witnessing only (1.9%, *n* = 187), victimization and witnessing (2.3%, *n* = 220), perpetration and witnessing (0.4%, *n* = 38), or involvement in all three roles (3.0%, *n* = 291).

Significant differences across cyberbullying involvement types were observed for gender and school level (*p* < 0.001), as well as for all individual-level cyber-risk indicators, including risky online behaviors, acceptance attitude toward cyberbullying, duration of digital activity, and exposure to harmful peers (*p* < 0.001). These patterns indicate that both sociodemographic characteristics and individual-level cyber-risk indicators vary systematically across distinct configurations of cyberbullying involvement.

### 3.2. Logistic Regression

Table 2 presents the results of multinomial logistic regression analyses examining associations between individual-level cyber-risk indicators and types of cyberbullying involvement, with the no-involvement group serving as the reference category.

School level differences were pronounced across involvement types. Compared with elementary school students, middle school students exhibited significantly lower odds of witnessing only (odds ratio [OR] = 0.43, 95% confidence interval [CI]: 0.30–0.63), perpetration–witnessing (OR = 0.32, 95% CI: 0.14–0.69), victimization–witnessing (OR = 0.52, 95% CI = 0.37–0.73), and involvement in all three roles (OR = 0.66, 95% CI: 0.49–0.89). High school students demonstrated consistently lower odds across all involvement types, including perpetration only (OR = 0.48, 95% CI: 0.36–0.65), victimization only (OR = 0.67, 05% CI: 0.58–0.78), witnessing only (OR = 0.23, 95% CI: 0.15–0.36), perpetration–victimization (OR = 0.34, 95% CI: 0.28–0.40), perpetration–witnessing (OR = 0.04, 95% CI: 0.01–0.19), victimization–witnessing (OR = 0.22, 95% CI: 0.14–0.33), and tri-role involvement (OR = 0.15, 95% CI: 0.10–0.23). These findings indicate that younger adolescents, particularly elementary school students, are at greater risk of both single and overlapping cyberbullying involvement.

Across all models, individual-level cyber-risk indicators were significantly associated with at least one form of cyberbullying involvement. Risky online behaviors were consistently linked to higher odds of involvement, with odds ratio ranging from 1.41 (95% CI: 1.05–1.88) for witnessing only to 3.65 (95% CI: 2.92–4.56) for tri-role involvement. Duration of digital activity was associated with increased odds of perpetration only, victimization only, perpetration–victimization, victimization–witnessing, and tri-role involvement, indicating that prolonged online exposure elevates the likelihood of both singular and overlapping roles.

In contrast, greater acceptance attitudes toward cyberbullying were associated with lower odds of victimization only (OR = 0.82, 95% CI: 0.74–0.91), suggesting a potential normalization effect that may reduce self-identification as a victim rather than actual exposure.

Finally, association with friends who engage in cyberbullying emerged as the strongest predict repr resents all involvement types. Adolescents with harmful peers showed substantially higher odds of cyberbullying involvement, with odds ratios ranging from 1.58 (95% CI: 1.28–1.95) for victimization only to 6.58 (95% CI: 5.07–8.55) for tri-role involvement. This pattern underscores the central role of peer environments in shaping not only cyberbullying exposure but also the accumulation of multiple involvement roles.

## 4. Discussion

The present study identified clear developmental differences in cyberbullying involvement across school levels. Compared with elementary school students, middle and high school students showed significantly lower odds of experiencing both single and overlapping forms of cyberbullying. This pattern suggests that cyberbullying involvement may peak during the early stages of digital socialization, when children first move from passive media use to interactive online communication. Notably, high school students exhibited consistently lower odds across nearly all involvement types, indicating a gradual decline in cyberbullying participation as adolescents mature and acquire greater digital self-regulation and social awareness.

Across all models, individual-level cyber-risk indicators showed robust associations with cyberbullying involvement across multiple role configurations. Importantly, the observed gradients in association strength across increasingly complex involvement patterns are descriptive and inferentially indirect, as the analytic approach was not designed to test direct pairwise differences between single-role and overlapping-role groups. Risky online behaviors and longer durations of digital activity were associated with increased odds of both singular and multi-role involvement, highlighting how cumulative exposure within online environments amplifies vulnerability. In contrast, greater acceptance of cyberbullying behaviors was associated with lower odds of victimization. This pattern may reflect normative desensitization or differential interpretation of harmful encounters rather than reduced exposures, as adolescents who perceive such behaviors as acceptable may be less likely to label or report their experiences as victimization. Alternatively, higher acceptance may co-occur with more provocative or risk-oriented online behaviors that alter peer interaction dynamics and targeting patterns. Together, these possibilities underscore the need to interpret this association with caution and highlight the importance of longitudinal research to disentangle attitudinal, behavioral, and peer-driven mechanisms. Moreover, these findings suggest that victimization does not uniformly escalate into perpetration, underscoring heterogeneity in adolescents’ responses to cyberbullying exposure. While victimization may function as a contextual trigger, escalation into perpetration appears contingent on individual susceptibility to disinhibited online responding. Digital environments characterized by reduced perceived accountability, attenuated social cues, and weakened self-regulatory control may lower thresholds for retaliatory or reactive aggression following victimization. In this context, cyber-risk profiles may reflect underlying differences in regulatory capacity and normative interpretations of online interaction, rather than exposure alone.

Building on the witnessing-related patterns, peer influence emerged as the strongest and most consistent correlate of cyberbullying involvement across all role configurations. Association with peers who engage in cyberbullying substantially increased the likelihood of involvement not only in singular roles but also in overlapping patterns, underscoring the central role of peer networks in shaping online behavior. This finding suggests that cyberbullying is embedded within shared social contexts rather than driven solely by individual characteristics or online exposure. Accordingly, prevention efforts may be most effective when they extend beyond individual-level interventions to address group norms and bystander dynamics. In particular, interventions that empower witnesses, promote prosocial digital norms, and foster collective responsibility within peer networks may be especially effective in disrupting recurrent cycles of cyberbullying.

Our findings reflect the current Korean context, in which early and intensive engagement with digital platforms has made cyberbullying a salient risk during childhood. Recent national data underscore the urgency of early intervention in Korea, as cyberbullying prevalence among elementary school students continues to rise, encompassing perpetration, victimization, and witnessing [74]. Elementary school students often exhibit lower levels of digital literacy [75], weaker self-regulatory capacities and limited experience navigating online social norms [76], rendering them more vulnerable to impulsive [77], imitative, or uncritical engagement in cyber interactions [78]. As adolescents transition into middle and high school, gains in cognitive maturity and executive functioning, enhanced social perspective-taking, and greater exposure to digital citizenship education likely contribute to the observed decline in cyberbullying involvement [39]. At the same time, this developmental pattern may partially reflect measurement limitations, as the survey items primarily capture overt forms of cyber bullying (e.g., direct insult), which may not fully encompass more subtle, relational, or context-dependent behaviors—such as social exclusion or indirect targeting—that are more characteristic of older adolescents. Accordingly, age-related differences should be interpreted with caution. The strong association between risky online behaviors and multi-role involvement further indicates that effective prevention should move beyond punitive responses and prioritize developmentally informed, skill-building interventions that foster empathy, communication, and online self-regulation.

This trend coincides with early smartphone adoption and increased engagement with social media and online gaming platforms, which facilitate rapid peer interaction and accelerate the escalation of online conflicts [79]. Early adolescence is also a critical period for online identity formation, during which negative or unstable cyber identities have been linked to early cyber delinquency and aggressive online behavior [80]. Together, these dynamics underscore the importance of integrating online identity education into mandatory, school-based violence prevention curricula during the early years of schooling.

In response to these challenges, Korea has implemented several large-scale initiatives aimed at preventing cyberbullying. Notably, the Ministry of Education’s Cyber Oullim Program emphasizes empathy, communication, emotional regulation, and conflict resolution across developmental stages [81]. Evaluation studies have reported meaningful improvements in emotional competencies and bullying prevention outcomes, particularly among elementary school students [82,83,84,85], although effects on classroom cohesion have been mixed [86,87]. Complementing these governmental efforts, non-profit initiatives such as the Blue Elephant Cyberbullying Prevention Program have demonstrated reductions in victimization and increases in defensive and bystander behaviors [88,89]. Despite these positive outcomes, existing programs remain fragmented, and few offer a systematic framework for online identity development. Establishing a standardized national curriculum that integrates identity formation with digital literacy may therefore enhance the long-term effectiveness and coherence of prevention efforts.

Beyond individual programs, our study also highlights broader implications of online coexistence in children’s daily lives. Structural features of digital environments—such as anonymity, continuous connectivity, and rapid interaction—can intensify peer influence, prolong screen time, and normalize exposure to harmful content [90]. In recognition of these risks, the Korean government expanded institutional capacity in 2024 by deploying dedicated investigators to support schools in addressing cyberbullying and other forms of school violence [91]. While this initiative strengthens professional oversight and crisis response, concerns remain regarding potential over-policing and unintended negative effects on school climate. Accordingly, a balanced approach that combines institutional support with preventive education and empathy-based interventions is essential for sustaining supportive and inclusive digital learning environments.

These findings should also be interpreted within Korea’s broader sociocultural and institutional context, particularly in light of our results emphasizing the central role of peer networks and witnesses in cyberbullying dynamics. Collective norms that prioritize social harmony and conformity may already discourage reporting or active bystander intervention, especially among witnesses, while hierarchical social structures can reinforce power asymmetries in online interactions [92,93,94,95]. Within this context, Korea’s highly competitive educational environment may further intensify stress-related aggression in cyberspace [96,97]. Reflecting growing societal concern, the Korean Ministry of Education has recently implemented a stringent policy linking records of school violence—including cyberbullying adjudicated at Level 4 or higher by the School Violence Countermeasures Committee—to mandatory consideration in all college admissions beginning in 2026 [98]. While this policy underscores the seriousness of cyberbullying [99], its punitive and high-stakes nature may also carry unintended consequences, such as discouraging disclosure or pushing harmful behaviors into less visible forms, particularly among witnesses who are critical to early detection and peer intervention. In contrast, our findings highlight the importance of prevention strategies that strengthen empathy, peer responsibility, and supportive bystander norms. Rather than relying solely on top-down deterrence, culturally responsive approaches that empower witnesses and foster prosocial digital climates may be more effective in reducing cyberbullying while avoiding long-term educational consequences for youth.

This study contributes to the literature in four important ways. First, it draws on a large, nationally representative dataset spanning elementary to high school students. Second, it extends beyond the traditional perpetrator-victim dichotomy by explicitly incorporating witnessing. Third, it identifies eight mutually exclusive involvement patterns, allowing for nuanced comparisons of overlapping roles. Fourth, it demonstrates that individual-level cyber-risk indicators—encompassing online behaviors, peer environments, and digital engagement—play a prominent role in differentiating patterns of cyberbullying experiences.

Several limitations should be acknowledged. First, the reliance on self-reported socioeconomic measures may introduce reporting bias; future research would benefit from incorporating objective indicators. Second, the use of single-item measure limited measurement specificity across several constructs. In particular, the single-item measure of internet use did not distinguish between different activity types (e.g., educational, social, or informational), and the single-item, dichotomized indicators for witnessing cyberbullying and exposure to harmful peers constrained measurement depth; notably, binary coding of the “harmful friend” variable obscured potential gradient or cumulative effects implied by the original ordinal scale, precluding examination of dose–response relationships related to peer context. Third, the internal consistency of the risky online behaviors scale was relatively low. This likely reflects the heterogeneous nature of the items, which capture a broad range of distinct online risk behaviors rather than a narrowly defined unidimensional construct. Although such reliability levels may be acceptable for exploratory indices, the lower internal consistency may attenuate effect estimates and weaken inferences drawn from this specific indicator. Accordingly, findings related to risky online behaviors should be interpreted with caution. Fourth, although the original survey items assessed cyberbullying experiences using ordinal frequency scales, the national survey data exhibited low proportions of high-frequency responses. Retaining the full original scale therefore risked unstable estimates due to response clustering in lower-frequency categories. Accordingly, responses were dichotomized to enhance interpretability, ensure comparability with national policy statistics, and improve the robustness of involvement-type classification. However, this decision represents a meaningful methodological trade-off. By converting frequency-based measures into binary indicators (any vs. none), the analysis necessarily lost information regarding the frequency and intensity of cyberbullying experiences. As a result, individuals classified within the same role category may have differed substantially in their exposure levels ranging from isolated incidents to repeated or chronic experiences. While dichotomization facilitated the identification of clear and mutually exclusive role typologies, it precluded examination of dose–response relationships and chronicity effects that may be important for understanding differential outcomes. Future studies should consider alternative modeling strategies (e.g., nested or direct contrast models) to formally test differentiation between single-role and overlapping-role involvement patterns, while also capturing both role typologies and variation in the frequency or intensity of cyberbullying exposure.

## 5. Conclusions

The current findings highlight the critical need for developmentally targeted cyberbullying prevention, particularly during early adolescence in Korea. The rising prevalence of cyberbullying among elementary school students highlights the urgency of early, comprehensive interventions that integrate digital literacy, online identity education, and peer-focused strategies. While governmental and non-profit initiatives show considerable promise, their effectiveness will depend on sustained coordination, systematic evaluation, and adaptation to developmental needs. Collaborative efforts among policymakers, educators, families, and communities are essential to fostering safer, more supportive online environments for children and adolescents.

## Figures and Tables

**Table 1:** Descriptive statistics of study variables in overall sample and by the types of cyberbullying experiences (*N* = 9693).

	Cyberbullying Types									
	Overall Sample (*n* = 9693)	Perpetration Only (*n* = 371, 3.8%)	Victimization Only (*n* = 1793, 18.5%)	Witnessing Only (*n* = 187, 1.9%)	Perpetration and Victimization Only (*n* = 1299, 13.4%)	Perpetration and Witnessing Only (*n* = 38, 0.4%)	Victimization and Witnessing Only (*n* = 220, 2.3%)	All Involved Only (*n* = 291, 3.0%)	None Involved Only (*n* = 5494, 56.7%)	*x*^2^/*F*
Gender, *n* (%)										
male	4820 (49.7)	241 (65.0)	896 (50.0)	108 (57.8)	869 (66.9)	22 (57.9)	104 (47.3)	184 (63.2)	2396 (43.6)	*p* < 0.001 ^a^
female	4873 (50.3)	130 (35.0)	897 (50.0)	79 (42.2)	430 (33.1)	16 (42.1)	116 (52.7)	107 (36.8)	3098 (56.4)	
School years, *n* (%)										
Elementary school	3401 (35.1)	127 (34.2)	600 (33.5)	110 (58.8)	462 (35.6)	24 (63.2)	117 (53.2)	132 (45.4)	1829 (33.3)	*p* < 0.001 ^a^
Middle school	3229 (33.3)	144 (38.8)	631 (35.2)	47 (25.1)	556 (42.8)	12 (31.6)	66 (30.0)	122 (41.9)	1651 (30.1)	
High school	3063 (31.6)	100 (27.0)	562 (31.3)	30 (16.0)	281 (21.6)	2 (5.3)	37 (16.8)	37 (12.7)	2014 (36.7)	
Subjective academic achievement M (SD)	3.36 (1.17)	3.30 (1.20)	3.38 (1.16)	3.44 (1.19)	3.34 (1.18)	3.42 (1.26)	3.47 (1.18)	3.44 (1.18)	3.35 (1.17)	-
Perceived financial situation M (SD)	3.38 (0.89)	3.29 (0.89)	3.37 (0.87)	3.49 (1.05)	3.36 (0.90)	3.42 (1.11)	3.37 (1.00)	3.48 (0.99)	3.39 (0.88)	-
Individual-level cyber-risk indicators, M (SD)										
Risky behavior	1.57 (0.49)	1.70 (0.49)	1.60 (0.44)	1.57 (0.52)	1.75 (0.48)	1.88 (0.62)	1.63 (0.48)	1.96 (0.59)	1.48 (0.47)	*p* < 0.001 ^b^
Awareness of online behavior	1.34 (0.57)	1.42 (0.57)	1.29 (0.47)	1.46 (0.76)	1.39 (0.46)	1.46 (0.59)	1.35 (0.58)	1.54 (0.64)	1.32 (0.61)	*p* < 0.001 ^b^
Duration of digital activity	3.45 (1.22)	3.66 (1.19)	3.54 (1.16)	3.05 (1.34)	3.75 (1.12)	3.45 (1.29)	3.41 (1.27)	3.64 (1.18)	3.34 (1.25)	*p* < 0.001 ^b^
Harmful friend	1.09 (0.37)	1.11 (0.42)	1.06 (0.27)	1.23 (0.54)	1.22 (0.56)	1.47 (0.92)	1.29 (0.55)	1.55 (0.80)	1.03 (0.21)	*p* < 0.001 ^b^

Note. M = mean; SD = standard deviation. ^a^: calculated chi-square test; ^b^: calculated using analysis of variance.

**Table 2:** Estimates of association of cyberbullying involvement (vs. None involved) with cyberspace behaviors (*N* = 9693).

	Cyberbullying Types (*Ref*. None Involved)						
	Perpetration Only (*n* = 371)	Victimization Only (*n* = 1793)	Witnessing Only (*n* = 187)	Perpetration and Victimization Only (*n* = 1299)	Perpetration and Witnessing Only (*n* = 38)	Victimization and Witnessing Only (*n* = 220)	All Involved Only (*n* = 291)
	*OR* (95% CI)						
Gender	**2.17 (1.73–2.73)** ***	**1.25 (1.12–1.40)** ***	**1.53 (1.12–2.07)** **	**2.27 (1.98–2.60)** ***	1.35 (0.68–2.67)	1.01 (0.76–1.35)	**1.52 (1.15–2.01)** **
School years (*ref*. elementary)							
Middle school	0.88 (0.67–1.14)	0.96 (0.84–1.10)	**0.43 (0.30–0.63)** ***	0.90 (0.77–1.05)	**0.32 (0.14–0.69)** **	**0.52 (0.37–0.73)** ***	**0.66 (0.49–0.89)** **
High school	**0.48 (0.36–0.65)** ***	**0.67 (0.58–0.78)** ***	**0.23 (0.15–0.36)** ***	**0.34 (0.28–0.40)** ***	**0.04 (0.01–0.19)** ***	**0.22 (0.14–0.33)** ***	**0.15 (0.10–0.23)** ***
Subjective academic achievement	1.03 (0.93–1.14)	**1.05 (1.00–1.11)** *	0.94 (0.81–1.08)	1.04 (0.98–1.11)	1.02 (0.74–1.41)	1.08 (0.94–1.24)	1.09 (0.96–1.24)
Perceived financial situation	**0.87 (0.76–0.99)** *	0.95 (0.89–1.01)	1.02 (0.85–1.22)	0.93 (0.86–1.01)	0.90 (0.61–1.33)	0.88 (0.75–1.04)	0.98 (0.84–1.14)
Individual-level cyber-risk indicators							
Risky behavior	**1.82 (1.49–2.22)** ***	**1.64 (1.46–1.84)** ***	**1.41 (1.05–1.88)** *	**2.49 (2.18–2.85)** ***	**2.94 (1.77–4.89)** ***	**1.81 (1.38–2.37)** ***	**3.65 (2.92–4.56)** ***
Cyberbullying accepting attitude	1.08 (0.92–1.28)	**0.82 (0.74–0.91)** ***	1.15 (0.94–1.40)	0.96 (0.86–1.08)	1.08 (0.67–1.75)	0.95 (0.75–1.19)	1.20 (1.00–1.45)
Duration of digital activity	**1.31 (1.19–1.44)** ***	**1.18 (1.12–1.24)** ***	0.97 (0.85–1.10)	**1.44 (1.36–1.53)** ***	1.31 (0.98–1.74)	**1.22 (1.08–1.38)** **	**1.37 (1.22–1.54)** ***
Harmful friend	**1.79 (1.35–2.39)** ***	**1.58 (1.28–1.95)** ***	**3.16 (2.35–4.24)** ***	**3.77 (3.08–4.63)** ***	**4.18 (2.69–6.50)** ***	**4.10 (3.09–5.43)** ***	**6.58 (5.07–8.55)** ***

Note. *OR* = odds ratio; CI = confidence interval. Subjective academic achievement: self-perceived academic performance. Subjective income: self-perceived financial status of the household. Bold represents statistically significant *p*-value at * *p* < 0.05, ** *p* < 0.01, *** *p* < 0.001.

## Data Availability

The data used in this study were obtained from previously collected sources and are not publicly available due to data protection and privacy restrictions.
